# A concealed history behind the disaster: Extremely rare presentations of metformin toxicity in a patient with body dysmorphic disorder

**DOI:** 10.1016/j.toxrep.2022.04.004

**Published:** 2022-04-12

**Authors:** Shokoufeh Hajsadeghi, Milad Gholizadeh Mesgarha, Arash Pour Mohammad, Ali Saberi Shahrbabaki, Aisa Talebi

**Affiliations:** a Research center for prevention of cardiovascular disease, Institute of endocrinology & metabolism, Iran University of Medical Sciences (IUMS), Tehran, Iran; b Faculty of Medicine, Iran University of Medical Sciences (IUMS), Tehran, Iran; c Faculty of Medicine, Shahid Beheshti University of Medical Sciences, Tehran, Iran

**Keywords:** Metformin, Metabolic acidosis, Cardiogenic shock, Megaloblastic anemia, Neuropathy, Pancytopenia, Hyponatremia, Body dysmorphic disorder, Hyperlactatemia, Lactic acidosis, Toxicity, Intoxication, Biguanide, Acute Kidney Injury

## Abstract

Metformin is a widely used anti-hyperglycemic agent with weight loss effect properties but besides its various utilities, despite being very rare, it has its characteristic toxicity and adverse effects when used in large doses and for the long-term or in patients with renal impairment. We presented here a case of a 36-year-old woman who developed several presentations with diverse features during three years comprising neuropathic symptoms, severe lactic acidosis, three episodes of cardiogenic shock, acute kidney injury, megaloblastic anemia, pancytopenia, and hyponatremia and did not receive a definite diagnosis after each presentation until when she inadvertently disclosed her abuse of extremely unusual doses of metformin during these three years with aim of weight reduction obsessively without knowing that her symptoms could pertain to metformin overdose. She was eventually diagnosed with a body dysmorphic disorder which led to unreasonable abuse of metformin pills that consequently caused its toxicity. Thereafter, with cease of metformin use and psychiatric treatment, her symptoms did not recur and she was doing well after one year of her last admission. Based on the review of the literature, this is the first case of metformin toxicity in a patient with body dysmorphic disorder who was affected with extremely rare features of this intoxication, nevertheless, every manifestation of the patient was discussed exhaustively according to the current and available medical literature.

## Highlights

• Metformin is an expedient blood glucose lowering agent with proposed weight loss effects.
• A 36-year-old women with body dysmorphic disorder (BDD) showed diverse presentations of extreme doses of metformin abuse.
• Metformin was the culprit for cardiogenic shock, lactic acidosis, megaloblastic anemia and pancytopenia.
• Obtaining diligent history is essential in BDD patients with such bewildering disease course.

## Graphical Abstract

**Figure:**
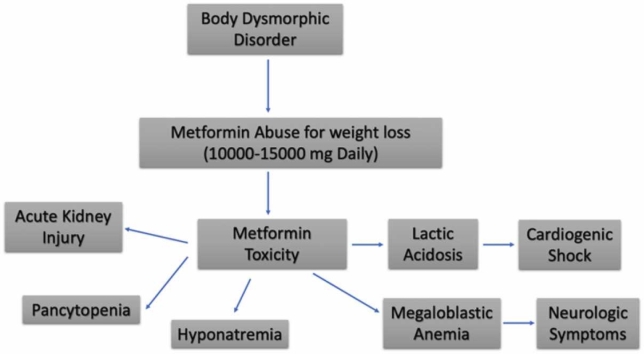

## 1 Introduction

Metformin is a broadly utilized biguanide drug which besides its main use as an anti-hyperglycemic agent, has various benefits [1]. One of them is weight loss-inducing through a number of mechanisms, though it remains obscure to employ metformin as a primary treatment for obesity and as a weight-loss drug [1], [2].

Apart from its utility, metformin has its own adverse effects including gastrointestinal problems, cobalamin deficiency, and rarely hemolytic anemia. In addition, despite its rarity, it has characteristic toxicity with metabolic acidosis and hyperlactatemia which can occur in two clinical settings: acute overdose and renal failure due to poor clearance of metformin [3].

Herein, we introduced a challenging case of a young woman who experienced very rare and various features of metformin toxicity in multiple episodes during three years following daily use of incredible doses of metformin pills without being diabetic or having suicidal ideation but with the purpose of weight loss obsessively and unusually, being unaware of this drug as a culprit for her symptoms and finally diagnosed with body dysmorphic disorder (BDD).

Based on the review of the literature, although two cases of patients with anorexia nervosa have been reported who encountered acute metformin intoxication when they used metformin with the aim of weight loss [4], [5], there are two distinct features in this case report including diversity and rarity of metformin toxicity presentations and this intoxication in a patient with underlying BDD. Accordingly, we discussed pathologic processes leading to these manifestations of metformin toxicity and possible causes of this drug abuse in BDD patients.

## 2 Case presentation

A 36-year-old woman presented to the emergency department of our hospital with acute dyspnea, nausea, and altered mental status. She was well until one week before presentation when she developed progressive malaise and weakness, followed by dyspnea and then confusion on the day of her admission. She reported no coughing, hemoptysis, chest pain, palpitations, night sweats, fevers, chills, or abdominal pain. Her medical history was notable for progressive paresthesia and weakness of left upper & left lower limbs which did not receive a definitive diagnosis and with a probable diagnosis of transverse myelitis, she was treated with corticosteroids three years ago. Another prominent medical history was two episodes, similar to the current presentation, which had occurred about two and a half and two years ago: the first one with a presentation of orthopnea and the second one with a loss of consciousness.

Cardiogenic shock was clinically diagnosed for both episodes in other centers and she was managed conservatively in ICU which resulted in her improvement. Upon her diagnostic workup following the second episode of cardiogenic shock in another health care center, cardiac magnetic resonance (CMR) revealed global hypokinesia prominently in the anterior wall, myocardial inflammation in the basal to the mid septal wall, subepicardial fibrosis in the anteroseptal wall, and reduced ejection fraction (EF) (35%).

Given the medical history of possible transverse myelitis, these two episodes of the cardiogenic shock of unknown cause with these CMR findings were assumed to be caused by autoimmune myocarditis and rheumatologic diseases were suspected, therefore the patient was put on immunosuppressive drugs. Based on the review of the patient’s medical records, in her follow-up echocardiography normal left ventricle function was noticed between these two episodes of cardiogenic shock.

She had taken cyclophosphamide and prednisolone but discontinued their consumption on her own four months before presentation.

Upon physical examination, the patient appeared confused and in respiratory distress. The respiratory rate was 30 breaths per minute but vital signs were otherwise normal. Other abnormal findings in the systemic examination were pale conjunctiva and bilateral basal crackles on the lung auscultation. On the second day of admission, the patient’s clinical condition deteriorated and her consciousness declined to the lethargic level. The oxygen saturation decreased to 70% while she was breathing ambient air, the blood pressure was 78/49 mm Hg, pulse rate was 128 beats per minute, and respiratory rate was 36 breaths per minute. Her peripheral pulses were weakly palpable.

Electrocardiogram showed sinus tachycardia and was otherwise normal. According to the aforementioned clinical status, the patient was transferred to the ICU, underwent mechanical ventilation, and norepinephrine and dopamine were started. Bedside echocardiography showed a severely reduced EF (20%) with normal right ventricle size and function and no evidence of pericardial effusion. Abnormal laboratory data at that time included hemoglobin level of 7.8 g per deciliter with the mean corpuscular volume 106.5 fl, potassium level 6.3 mmol per liter, phosphorus level 8.3 mmol per liter, Blood urea nitrogen level 103 mg per deciliter, and creatinine level 4.7 mg per deciliter. Arterial blood gas analysis showed a pH of 6.8, partial pressure of carbon dioxide of 27 mmHg, a bicarbonate level of 3 mmol per liter, and a serum lactate level of 15 mmol per liter. Accordingly, the patient underwent emergent hemodialysis twice with an interval of 48 h. Renal and color doppler ultrasound were unremarkable and comprehensive rheumatologic serology was inconclusive. In spite of the negative results of autoimmune serology tests and considering her past medical history, an autoimmune process like fulminant vasculitis was still suspected, consequently, intravenous immune globulin and methylprednisolone were administered.

On the fifth day of admission, transthoracic echocardiography revealed an EF of 15% and in consultation with the treatment team, given the patient’s youth and the development of unexplained and critical episodes of transient low ejection fractions, the patient became a candidate for an emergent left ventricle assist device or heart transplant. In the interim, pancytopenia with a white blood cell count of 3300 per microliter, a Hemoglobin level of 8.1 per deciliter, and a platelet level of 70,000 per microliter developed which caused cessation of the patient transfer for cardiac replacement therapy and implementation of bone marrow study which further revealed normal bone marrow aspiration and biopsy.

Surprisingly on the sixth day of hospital stay, the patient extubated herself, oxygen saturation markedly improved to the normal level, blood pressure increased to the normal range despite discontinuing treatment with vasopressors and other clinical signs and symptoms dramatically abated. Thereafter, she underwent a spiral chest CT scan which showed bilateral pleural effusion with cardiomegaly. She also underwent brain MRI which showed restricted and abnormal signal intensity in the bilateral external segment of globus pallidus. To investigate active vasculitis, a PET scan was performed and resulted normally. Follow-up echocardiography was performed in the second week of hospitalization which demonstrated an EF of 50% with normal left ventricle size and function. The patient was discharged home on prednisolone 75 mg per day and cyclophosphamide 50 mg twice a day.

After one month of discharge, the patient presented to the emergency department once more with confusion, nausea, vomiting, and malaise. On physical examination, she appeared confused but not in distress. Her vital signs were stable and only bilateral mild pitting edema of the lower extremities was noted on examination. In her laboratory data, a low sodium serum level (110 mmol per liter) and increased serum creatinine level (2 mg per deciliter) were notable; according to this hyponatremia and acute kidney injury (AKI), she was treated by hypertonic saline which led to clinical improvement. Transthoracic echocardiography was performed which showed a normal EF (55%). Other diagnostic work up comprising urine toxicology, serum level of vitamin B12 and folate was normal. Afterward, the patient was discharged in generally good condition.

After one week of the discharge, in an outpatient cardiology visit, the patient’s cardiologist measured her height and weight before performing follow-up echocardiography on a routine basis. Subsequently, the patient instantly asked her physician about her body habitus and while she was looking for an answer obsessively, her mother interrupted her and urged the physician to assist her in the prevention of abusive drug consumption. Upon further questioning from the patient, she disclosed that she has taken about 20–30 pills of metformin 500 mg daily since 3 years ago to lose weight.

**Fig. 1:**
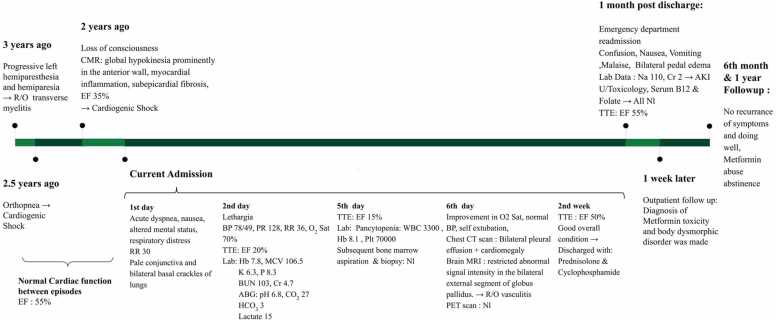
Temporal course of patient's multiple admissions and clinical findings. R/O: Rule Out, CMR: Cardiac Magnetic Resonance, TTE: Transthoracic Echocardiography, EF: Ejection Fraction, Nl: Normal, RR: Respiratory Rate, BP: Blood Pressure, PR: Pulse Rate, O_2_ Sat: O_2_ Saturation, Hb: Serum Hemoglobin, WBC: White Blood Cells, Plt: Platelets, MCV: Mean Corpuscular Volume, ABG: Arterial Blood Gas, AKI: Acute Kidney Injury.

Given this unraveled history, the diagnosis of multiple episodes of metformin toxicity which can justify the whole patient presentation’s picture throughout these three years was made and body dysmorphic disorder was suspected as an underlying cause. As a result, she was referred to a psychiatrist who confirmed this diagnosis and she was started on risperidone, fluoxetine, and amitriptyline besides psychotherapy. In her follow-up in 6 months and 1 year after the last admission, she reported that she discontinued abuse of metformin and was doing well without recurrence of symptoms, her follow-up echocardiography results were also normal (EF: 55%) and abnormal laboratory data has markedly improved including WBC of 7200, Hb 14.5 with MCV of 95, Plt 340000, BUN 21,Cr 1.7, Na 139 and normal VBG findings. The chronological sequence of patient's signs and symptoms during his several admissions have been depicted in Fig. 1.

## 3 Discussion

In essence, we presented a case of a young woman with an undiagnosed background of body dysmorphic disorder who manifested numerous but rare features of metformin toxicity owing to her pathological desire to lose weight by means of arbitrary consumption of large doses of metformin and those clinical features comprised recurrent cardiogenic shocks, hyponatremia, acute renal failure, neuropathy, megaloblastic anemia, and pancytopenia.

Considering cardiovascular involvement of metformin toxicity, the medical literature has scant information; nonetheless, reduced left ventricular function and decreased cardiac output, cardiogenic shock and hypotension, cardiac dysrhythmia, and ultimately cardiac arrest are reported consequences in patients affected by metformin intoxication [6], [7]. These cardiac toxicity features are mainly attributed to the non-hypoxic (type B) lactic acidosis caused by metformin overdose (so-called “MALA” or metformin-associated lactic acidosis) which leads to diminished myocardial contractility and myocardial injury [6], [7], [8]. Furthermore, our patient experienced three episodes of severely reduced EF but after each episode she had almost completely normal cardiac function upon follow-up echocardiography; the fact that further corroborates cardiovascular toxicity is assigned to MALA which is rapidly reversible without long-term adverse outcome with use of supportive treatment encompassing extracorporeal therapy such as hemodialysis that was done for our patient [9].

Our patient developed profound hyponatremia and its manifestation in her last presentation; although there is insufficient data over its prevalence and pathophysiology; metformin is considered one of the hypoglycemic agents which causes hyponatremia [10], [11]. It is postulated that metformin can fortify the antidiuretic hormone (ADH) effects [11], and in one study by Sua Kim et.al, metformin has been found to prompt aquaporin-2 (AQP2) protein upregulation through different vasopressin receptor 2 (V2R)-independent pathways [12]. This cited pathophysiology was discovered in animal experiments and not in clinical practice, nevertheless, we assume that the reason for this lack of confrontation is the consumption of the usual dose of metformin but given this uncommonly abuse of metformin in our case, this hyponatremia can be ascribed to metformin toxicity.

Considering normal baseline levels of creatinine, AKI was suspected for her serum creatinine rise in both last presentations to our hospital. Although prerenal AKI secondary to cardiogenic shock could be assumed for the first presentation of the patient to our hospital, for the second presentation with hyponatremia and normal cardiac function, it does not seem to be a reasonable justification. There is a complicated interaction between metformin, AKI, and lactic acidosis [13], although an increased incidence of AKI was observed in diabetic patients in a study by G.Cavoli et.al, S. Bell's study demonstrated that metformin does not augment the incidence of AKI [14], [15]. Despite this controversy, metabolic acidosis was found to be an independent risk factor for the development of AKI and hospital mortality in a study by J. Hu et.al [16]. As a result, we can conclude the patient’s acidemia could have a role in her renal injury but further comprehensive studies are required to prove this hypothesis.

In view of the patient’s neurologic symptoms and megaloblastic anemia, vitamin B12 deficiency was presumed as a cause. It is well-known that metformin can give rise to cobalamin deficiency dose-dependently in long-term use [17], [18], and we did not find any other underlying causes which could explain neurological involvement and macrocytic anemia of the patient. Notwithstanding that serum B12 level was normal once checked in our work-up, we regarded it as a false normal assay [19], and it would be rational to further investigate B12 deficiency via functional biomarker of vitamin B12 status including Methylmalonic acid and total homocysteine when the metformin toxicity was recognized as final diagnosis [18].

In light of the transient pancytopenia which persisted for only two days and resolved with the administration of few doses of hematopoietic growth factors and mainly with refinement of clinical status, we hypothesized three mechanisms: firstly the B12 deficiency which is capable to induce pancytopenia, although the acute and transient presentation of pancytopenia is not usual for this diagnosis [20], secondly the metformin toxicity itself; despite the lack of adequate knowledge about association between metformin and pancytopenia, there is evidence in mice that metformin can affect in a dose- and time-dependent manner on bone marrow-derived multipotent mesenchymal stromal cells (BMSCs) leading to inhibition of proliferation and abnormalities of their morphology and ultrastructure, therefore it can be postulated that a similar scenario can exists in human when very large doses of metformin are consumed in long-term [21]; and thirdly, even though we did not find a valid information, we supposed severe lactic acidosis itself could negatively influence bone marrow and caused its suppression.

These manifestations discussed above occurred in a patient who was eventually diagnosed with body dysmorphic disorder (BDD). BDD is classified as one of the obsessive-compulsive and related disorders according to the diagnostic and statistical manual of mental disorders 5 (DSM-5) and our patient was suspected of this disorder when she was seeking reassurance from her physician about her body shape [22]. BDD is a relatively prevalent psychiatric disorder; however, it is an underdiagnosed clinical condition [23]. Moreover, weight concerns are rather common in BDD patients [24]; thus it warrants consideration that when we confront a patient who uses weight loss medications excessively, think of a possibility of BDD in the background. Additionally, when we encounter BDD patients, reflect on their drug history particularly those leading to weight reduction. Similar attention should be given to patients with eating disorders (e.g. anorexia nervosa) who are susceptible to abuse of weight loss drugs incorporating metformin as there are also two reports of metformin toxicity following its abuse in patients with anorexia nervosa [4], [5].

## 4 Limitation of the study

This case report and literature review had two main limitations: Although the clinical course and the patient’s declaration of metformin abuse sufficiently corroborate that the cause of the whole picture of patient's presentation is metformin toxicity, there was no laboratory data to affirm the high serum metformin level. Another limitation was related to the patient’s past medical history which eventually was found to be the result of metformin intoxication but detailed history and disease course were not thoroughly available.

## 5 Conclusion

In brief, this case exemplified a long-term missed diagnosis in clinical practice indicating the key to the diagnosis was probing the concealed drug abuse upon taking a meticulous history, it provided unforeseen clinical presentations of the metformin intoxication which clinicians should be aware of and manifested that a probable psychiatric background (i.e. BDD) could exist behind weight loss drugs abuse which knowing it has crucial importance in patient’s management.

## Funding

We received no funding for this article.

## Author's contribution

Shokoufeh Hajsadeghi and Milad Gholizadeh Mesgarha were responsible for patient management and conceptualization; Milad Gholizadeh Mesgarha, Ali Saberi Shahrbabaki and Aisa Talebi were accountable for composing the initial draft and literature review; Arash Pour Mohammad made the diagram, final revisions and organized patient's follow-ups.

## Declaration of Competing Interest

The authors declare that they have no known competing financial interests or personal relationships that could have appeared to influence the work reported in this paper.

## Data Availability

Data sharing not applicable to this article as no datasets were generated or analysed during the current study.
